# Exploiting high-quality reconstruction image encryption strategy by optimized orthogonal compressive sensing

**DOI:** 10.1038/s41598-024-59277-z

**Published:** 2024-04-16

**Authors:** Heping Wen, Lincheng Yang, Chixin Bai, Yiting Lin, Tengyu Liu, Lei Chen, Yingchun Hu, Daojing He

**Affiliations:** 1 Zhongshan Institute, University of Electronic Science and Technology of China, Zhongshan, 528402 China; 2 School of Information and Communication Engineering, University of Electronic Science and Technology of China, Chengdu, 611731 China; 3 School of Computer Science and Technology, Harbin Institute of Technology (Shenzhen), Shenzhen, 518118 China; 4 GuangDong Engineering Technology Research Center of Cryptographic Product and System Evaluation, Shenzhen, 518118 China

**Keywords:** Frequency domain compression, Image encryption, Compressive sensing, Optimized orthogonal, Engineering, Information technology, Computer science

## Abstract

Compressive sensing is favored because it breaks through the constraints of Nyquist sampling law in signal reconstruction. However, the security defects of joint compression encryption and the problem of low quality of reconstructed image restoration need to be solved urgently. In view of this, this paper proposes a compressive sensing image encryption scheme based on optimized orthogonal measurement matrix. Utilizing a combination of DWT and OMP, along with chaos, the proposed scheme achieves high-security image encryption and superior quality in decryption reconstruction. Firstly, the orthogonal optimization method is used to improve the chaotic measurement matrix. Combined with Part Hadamard matrix, the measurement matrix with strong orthogonal characteristics is constructed by Kronecker product. Secondly, the original image is sparsely represented by DWT. Meanwhile, Arnold scrambling is used to disturb the correlation between its adjacent pixels. Following this, the image is compressed and measured in accordance with the principles of compressive sensing and obtain the intermediate image to be encrypted. Finally, the chaotic sequence generated based on 2D-LSCM is used to perform on odd-even interleaved diffusion and row-column permutation at bit-level to obtain the final ciphertext. The experimental results show that this scheme meets the cryptographic requirements of obfuscation, diffusion and avalanche effects, and also has a large key space, which is sufficient to resist brute-force cracking attacks. Based on the sparse and reconstruction algorithm of compressive sensing proposed in this paper, it has better image restoration quality than similar algorithms. Consequently, the compressive sensing image encryption scheme enhances both security and reconstruction quality, presenting promising applications in the evolving landscape of privacy protection for network big data.

## Introduction

In the wake of the rapid evolution of computer communication and network technologies, diverse forms of data and information are being disseminated through networks with increased frequency, broader reach, and heightened velocity^1–3^. The emergence of these requirements for information interchange, particularly in ensuring a more secure transmission environment, has been noteworthy^4–6^. As one of the most intuitive and common data types in information transmission, images contain a substantial amount of sensitive information^7–9^. Consequently, the utilization of image encryption techniques can effectively prevent the leakage of critical data during transmission^10–12^. Several encryption methods have been proposed to address this challenge, including thumbnail-preserving encryption^13–15^, biometric encoding^16–18^, Frequency domain encryption^19,20^, bit-plane encryption^21–23^, Fourier transformation^24–26^, and chaos theory^27–29^, among others^30,31^. In particular, chaotic algorithms have gained widespread acceptance in image encryption due to their unpredictability, pseudo-randomness, and high sensitivity to initial values^32–35^.

Taking a global perspective, numerous scholars have achieved a series of significant theoretical and practical advancements in the use of chaos for image encryption^36–39^. Simultaneously, the field of compressive sensing theory has garnered favour among many cryptography experts due to its groundbreaking performance in signal sampling^40–42^. In 2021^43^, introduced an image encryption scheme based on four-winged hyperchaotic system, combined with compressive sensing and DNA encoding, effectively reduced the transmission cost. In 2022^44^, presented an image encryption approach that utilizes SHA-3 and an asymmetric key system. This method employs the RSA algorithm to encrypt the plaintext key and disclose the corresponding ciphertext key, avoiding the problem of additional transmission of the key in the channel. In 2023^45^, presented a secure and efficient image encryption approach that integrates parallel compression sensing with secret sharing. This method attains network system security and availability even with resource constraints. Within the ongoing exploration of compression-aware chaotic image encryption, the efficacy of chaotic sum algorithms significantly influences the security and efficiency of cryptographic systems. In contrast, most of the existing algorithms only obtain snowflake ciphertext images by scrambling at the pixel level or 2-bit, which has a coarse granularity and is susceptible to attacks from third parties. It is imperative and urgent to explore an image encryption algorithm with finer encryption granularity that utilizes chaotic mapping constructs to resist various illegal attacks.

This paper introduces a optimized orthogonal compressive sensing image encryption scheme based on 2D-LSCM. Firstly, the scheme adopts Discrete Wavelet Transform to sparsify the original image and performs Arnold scrambling on the sparse image to increase the uncorrelation between its neighbouring pixels. Secondly, based on the theory of CS, an optimized orthogonal measurement matrix is constructed by using the Kronecker product, a part Hadamard matrix and an optimized processed chaotic sequence to measure the sparse image and obtain the compressed measured matrix. Finally, odd-even interleaved diffusion and bit-level permutation is used for the measured matrix to obtain the final ciphertext image.

The main contributions of this paper are as follows:

- This paper proposes a security-enhanced, high-performance integrated image encryption scheme combining compressive sensing. Compared with most of the similar spatial domain based encryption schemes, it has lower computational complexity and improves the efficiency and security of encryption.
- A construction method for a compressive sensing measurement matrix is proposed. This method incorporates a plaintext correlation mechanism, ensuring that the measurement matrix possesses orthogonal characteristics. The orthogonal features of the measurement matrix contribute to improved reconstruction quality following decryption.
- In the image encryption algorithm, a chaotic key stream with plaintext correlation is generated. This key stream is utilized to control odd-even interleaved diffusion and row-column permutation operations at bit-level for encrypting the plaintext image. This approach offers a high granularity of encryption, effectively defending against chosen plaintext attack.
- Focusing on the sparse representation method and reconstruction algorithm of compressive sensing, this paper analyzes the impact of various parameters on recovery quality and selects the optimized combination scheme. Experimental results validate the superiority of the proposed scheme.

The organization of the remaining sections of this paper is as follows: Section "Related theory" provides a brief introduction to chaotic systems and the compressive sensing algorithm. Section "Image encryption and decryption scheme" presents the encryption algorithm designed in this paper. Section Simulation results and performance analysis offers experimental and simulation results. The final part concludes the paper.

## Related theory

### The used chaotic system

#### 2D-LSCM map

2D-LSCM is derived from two existing 1D chaotic mappings, namely the Logistic mapping and the Sine mapping^46^. The mapping diagrams are defined as follows:

1 $$\begin{aligned} {\left\{ \begin{array}{ll} {{x}_{i+1}}=\sin (\pi (4\theta {{x}_{i}}(1-{{x}_{i}})+(1-\theta )\sin (\pi {{y}_{i}}))) \\ {{y}_{i+1}}=\sin (\pi (4\theta {{y}_{i}}(1-{{y}_{i}})+(1-\theta )\sin (\pi {{x}_{i+1}}))) \\ \end{array}\right. } \end{aligned}$$

where $\theta$ denotes the control parameter, $\theta \in [0,1]$. The definition reveals the generation process, individual Logistic mapping and Sine mapping have been confirmed to have drawbacks such as simple behaviors and fragile chaotic intervals. Whereas 2D-LSCM couples both of these mappings and extends the dimension from 1D to 2D after performing a sinusoidal transformation on the coupling result. By this way, the complexity of Logistic mapping and Sinusoidal mapping can be fully mixed to obtain complex chaotic behavior.

#### 0-1 test results of chaos

**Figure 1:**
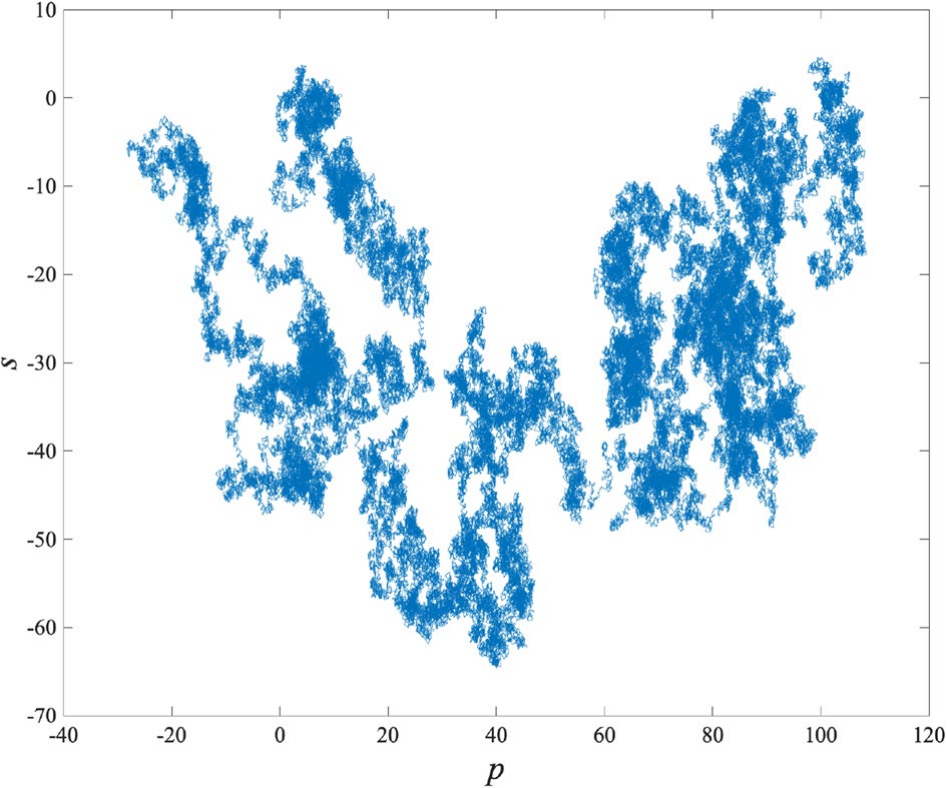
The Gottwald Melbourne 0-1 test of 2D-LSCM.

The Gottwald Melbourne 0-1 test serves as a computational instrument for determining parameters in close proximity to 0 or 1, facilitating a precise differentiation between regular and chaotic motion. In our investigation, the $0-1$ Gottwald Melbourne test was employed to generate 10,000 outcomes, demonstrating an average value of 0.9979. This notable result underscores the exceptional performance exhibited by the chaotic system. The graphical representation of the test outcomes is presented in Fig. 1.

### Compressive sensing

Donoho et al. proposed a novel signal sampling technique, where compression of the data is accomplished at the same time as sampling, named compressive sensing. The rationale is that if the signal is sparse, it can be accurately reconstructed by solving an optimization problem with a much smaller number of samples than required by the Nyquist sampling theorem.

**Figure 2:**
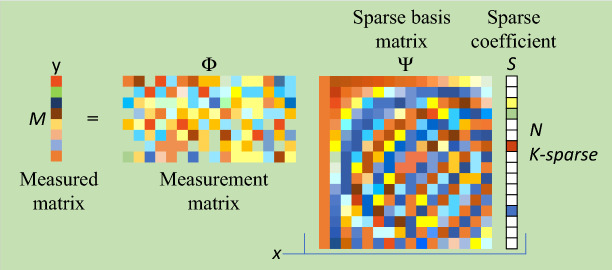
The schematic diagram of the compressive sensing process.

In compressive sensing, whether the signal has sparse properties is a prerequisite for judging whether the signal can be reconstructed accurately. Except for a few naturally sparse signals, most signals need to be represented sparsely on some sparse basis, described as $x=\Psi s$, where $\Psi$ is the sparse basis matrix, and *s* is the sparse coefficient. Assuming the signal *x* to be processed is either naturally sparse or can be sparsely represented (with size of $N\times 1$), the measurement process can be shown in Fig. 2 and expressed as:

2 $$\begin{aligned} y=\Phi x=\Phi \Psi s=As \end{aligned}$$

where $\Psi$ denotes the measurement matrix, utilized to project the high-dimensional signal *x* into a low-dimensional space, with size of $M\times N(M<N)$. And *y* denotes the measured matrix, with size of $M\times 1$. *A* denotes the sensing matrix, which is the product of the measurement matrix and the sparse basis matrix, and it can be represented as $A=\Phi \Psi$.

In compressive sensing theory, another crucial criterion for determining whether a signal can be reconstructed is assessing whether the sensing matrix satisfies the Restricted Isometry Property (RIP). If it does, the signal can be overwhelmingly reconstructed by solving the following convex optimization problem:

3 $$\begin{aligned} \min {{\left\| s \right\| }_{1}}\text { s}.\text {t}. \;y=\Phi \Psi s \end{aligned}$$

where $\min {{\left\| s \right\| }_{1}}$ denotes the ${{l}_{1}}$ norm of vector *s*. Orthogonal Matching Pursuit (OMP) and Basis Pursuit (BP) are both practical algorithms for solving such problems.

### Arnold map

**Figure 3:**
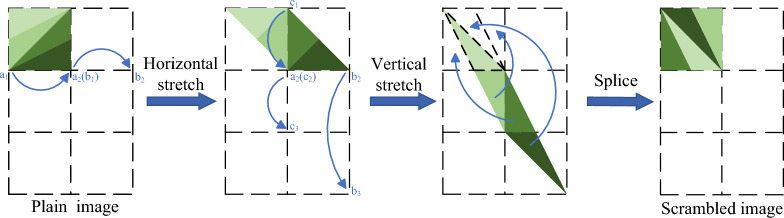
The geometric interpretation of Arnold Map.

Arnold mapping is widely used in image processing as a fast and effective scrambling method. It can be described as a stretching, folding and stitching process for a two-dimensional image matrix, the geometric interpretation of this process is shown in Fig. 3. For a image of size $M\times N$, the definition of Arnold map can be characterised by the following formula:

4 $$\begin{aligned} \left[ \begin{matrix} {{x}_{n+1}} \\ {{y}_{n+1}} \\ \end{matrix} \right] =\left[ \begin{matrix} 1 &{} b \\ a &{} ab+1 \\ \end{matrix} \right] \left[ \begin{matrix} {{x}_{n}} \\ {{y}_{n}} \\ \end{matrix} \right] \bmod N \end{aligned}$$

where ${{x}_{n}},{{y}_{n}}$ denote the original image pixel position, ${{x}_{n+1}},{{y}_{n+1}}$ denote the scrambled pixel position, *a*, *b* denote the mapping parameters.

Arnold mapping exhibits periodicity, with the period depending on the image dimensions, which implies that repeated permutations of an image can be reverted to the original image. For a square image of size $N\times N$, it can also be restored using the inverse transformation formula. The inverse Arnold mapping formula is as follows:

5 $$\begin{aligned} \left[ \begin{matrix} {{x}_{n}} \\ {{y}_{n}} \\ \end{matrix} \right] =\left[ \begin{matrix} ab+1 &{} -b \\ -a &{} 1 \\ \end{matrix} \right] \left[ \begin{matrix} {{x}_{n+1}} \\ {{y}_{n+1}} \\ \end{matrix} \right] \bmod N \end{aligned}$$

In image encryption algorithms combined with compressive sensing, it is crucial to perturb the image and reduce the correlation between adjacent pixels before measurement. This step can significantly enhance the reconstruction quality of the image.

## Image encryption and decryption scheme

**Figure 4:**
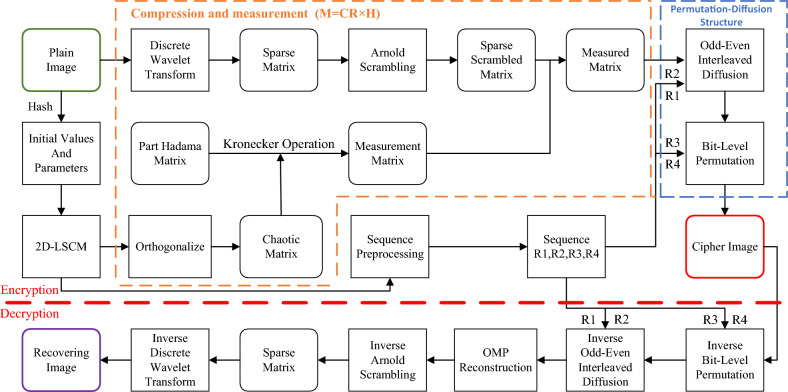
The overall schematic of the encryption and decryption scheme.

The proposed bit-level encryption scheme based on compressive sensing in this paper comprises two primary modules: compression measurement and the encryption of the digital format image. Firstly, the Discrete Wavelet Transform is used to convert the original image from the spatial domain to the frequency domain for sparse representation. After Arnold scrambling, the sparse image is compressed and measured to obtain the intermediate image to be encrypted. Finally, the chaotic sequence generated based on 2D-LSCM is used to encrypt the image with odd-even interleaved diffusion and row-column permutation at bit-level to obtain the final ciphertext image. The overall schematic of the scheme is shown in Fig. 4.

### Generation of the chaotic sequence and preprocessing

The key utilized in this algorithm is derived from the original image’s feature values obtained through a hash function. After preprocessing, these values are substituted into the chaotic system to generate the four necessary chaotic sequences.

*Step 1*: Extraction of image feature values

Use the hash SHA-256 to read the image feature value, which consists of a fixed 64-bit length of the hexadecimal number, and in order every four digits in a group to decimal representation, denoted as $K=\{{{k}_{1}},{{k}_{2}},......,{{k}_{16}}\}$. Depending on the value of ${k}_{5}$, different arrangement methods are chosen to surround these 16 numbers in order into a square matrix of size $4\times 4$.

*Step 2*: Preprocessing of image feature values

**Figure 5:**
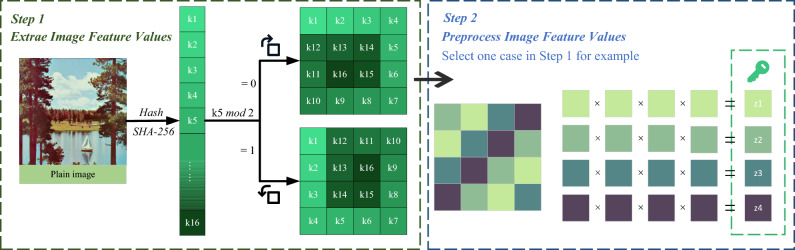
Schematic diagram of image feature values extraction and preprocessing.

For the obtained $4\times 4$ matrix, according to certain rules, each four-number group is processed and an initial key is generated. There are four groups, and the initial keys obtained are denoted as ${{z}_{1}},{{z}_{2}},{{z}_{3}},{{z}_{4}}$. The specific processing methods and schematics are shown in Fig. 5.

*Step 3*: Key perturbation and chaotic initialization generation

The initial value of the key is perturbed using Eq. (6) and ensures that its range falls between [0,1] as the initial parameter of the chaotic system.

6 $$\begin{aligned} ke{{y}_{i}}=\frac{{{z}_{i}}}{{{10}^{len\_{{z}_{i}}}}} \end{aligned}$$

where $ke{{y}_{i}}$ denotes the initial values of chaotic system, $len\_{{z}_{i}}$ denotes the length of ${{z}_{i}}$, $i=\{1,2,3,4\}$.

*Step 4*: The preprocessing of the chaotic sequences

By selecting any two combinations from $ke{{y}_{1}}$, $ke{{y}_{2}}$, $ke{{y}_{3}}$ and $ke{{y}_{4}}$, there are $C_{4}^{2}=6$ different possible combinations. Choosing any four of these combinations and substituting them into the 2D-LSCM chaotic system for iteration, while discarding the initial 1000 values, results in four distinct pseudorandom sequences denoted as $R_{1}$, $R_{2}$, $R_{3}$, $R_{4}$ respectively. The sequences $R_{1}$ and $R_{2}$ are used for odd-even interleaved diffusion. $R_{3}$ and $R_{4}$ are used to generate chaotic sort indexes for row-column permutation. The specific processing methods are as follows:

7 $$\begin{aligned} {\left\{ \begin{array}{ll} {{R}_{1}}=abs({{R}_{1}}\times {{10}^{15}}) \bmod 256 \\ {{R}_{2}}=abs({{R}_{2}}\times {{10}^{15}})\bmod 256 \\ {{R}_{3}}=abs({{R}_{3}}\times {{10}^{15}})\bmod 1 \\ {{R}_{4}}=abs({{R}_{4}}\times {{10}^{15}})\bmod 1 \\ \end{array}\right. } \end{aligned}$$

where $a \bmod b$ denotes the remainder of *a* over *b*, $\bmod$ 1 denotes that only take valid numbers after the decimal point.

### Generation of measurement matrix

In the compressive sensing based image encryption scheme, the measurement matrix is also transmitted to the receiver as one of the keys. To reduce the storage burden and transmission bandwidth required during transmission, this scheme uses Kronecker product operations to construct the measurement matrix. If two matrices of sizes $q\times p$ and $u\times v$ are both linearly independent low-dimensional orthogonal matrices, *a* linearly independent high-dimensional orthogonal matrix of size $uq\times pv$ can be obtained through the Kronecker product. The specific construction process is as follows:

*Step 1*: Arbitrarily select a set of keys and substitute them into the 2D-LSCM for $\text {4}\times \text {4}\times n+1000$ iterations, and discard the first 1000 values to obtain two sequences *X* and *Y* of length $4\times 4\times n$, where *n* is the sampling interval.

*Step 2*: Disturb sequence *X* and sequence *Y* to obtain a new sequence *Z*, as follows:

8 $$\begin{aligned} {{Z}_{i}}=\frac{M\times {{X}_{i}}+W\times {{Y}_{i}}}{2\times (M+W)} \end{aligned}$$

where $i=[1,2,3,...,4\times 4\times n]$, *M* denotes the height of the compressed image, *W* denotes the width of the compressed image.

*Step 3*: Arrange and reconstruct the obtained sequences in the following way:

9 $$\begin{aligned} A=orth\left( \sqrt{\frac{2}{M}}\times \left( \begin{matrix} {{Z}_{1+0\times n}} &{} {{Z}_{1+4\times n}} &{} {{Z}_{1+8\times n}} &{} {{Z}_{1+12\times n}} \\ {{Z}_{1+1\times n}} &{} {{Z}_{1+5\times n}} &{} {{Z}_{1+9\times n}} &{} {{Z}_{1+13\times n}} \\ {{Z}_{1+2\times n}} &{} {{Z}_{1+6\times n}} &{} {{Z}_{1+10\times n}} &{} {{Z}_{1+14\times n}} \\ {{Z}_{1+3\times n}} &{} {{Z}_{1+7\times n}} &{} {{Z}_{1+11\times n}} &{} {{Z}_{1+15\times n}} \\ \end{matrix} \right) \right) \end{aligned}$$

where $orth(\bullet )$ denotes orthogonalization. The pseudo-code for the orthogonalization function is given in algorithm 1.

**Algorithm 1:**
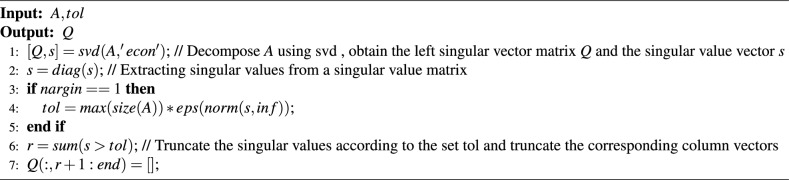
Orth.

*Step 4*: Perform Kronecker product operation on matrix $A\in {{\mathbb {R}}^{4\times 4}}$ and Part Hadama Matrix $B\in {{\mathbb {R}}^{\frac{M}{4}\times \frac{W}{4}}}$ to obtain the measurement matrix $\Phi \in {{\mathbb {R}}^{M\times W}}$.

10 $$\begin{aligned} \Phi =A\otimes B=\left( \begin{matrix} {{B}_{11}}A &{} {{B}_{12}}A &{} \cdots &{} {{B}_{1\frac{W}{4}}}A \\ {{B}_{21}}A &{} {{B}_{22}}A &{} \cdots &{} {{B}_{2\frac{W}{4}}}A \\ \vdots &{} \vdots &{} \ddots &{} \vdots \\ {{B}_{\frac{M}{4}1}}A &{} {{B}_{\frac{M}{4}2}}A &{} \cdots &{} {{B}_{\frac{M}{4}\frac{W}{4}}}A \\ \end{matrix} \right) \end{aligned}$$

The high-dimensional measurement matrix constructed through Kronecker product still maintains the non-correlation and orthogonality properties of the original matrices, which has been confirmed as an equivalent condition for *RIP*. To ensure the quality of reconstruction, the measurement matrix, such as Gaussian matrix, will be all sent to the decrypting party as a key under the traditional scheme, compared with the measurement matrix constructed by the method in this paper, the size of the matrix that needs to be transmitted additionally is only 6.25$\%$ of the traditional one.

### Encryption step

This paper uses a grayscale image as an example to illustrate the encryption process. For colour images, the encryption can be performed separately on the *R*, *G*, *B* channels. Assuming the input is an original image *P* with size $H$ $\times$ *W*, the specific compression measurement and encryption process are as follows:

*Step 1*: Sparsify the original image.

Using the Discrete Wavelet Transform, the original image is sparsely represented in the wavelet domain from the spatial domain, and obtain a sparse image of size $H \times W$. Additionally, in order to enhance the sparse characteristics, the pixel values in the sparse image are set to 0 if they are smaller than the threshold value.

*Step 2*: Scramble the sparse image.

In order to enhance the compression-aware reconstruction effect, this paper adopts Arnold scrambling to reduce the correlation between neighbouring pixels of the sparse image, and obtains the scrambled sparse image $P_{DWT}^{'}$. Let the parameter $a=b=1$ in Eq. (11), then the scrambling process can be described as follows:

11 $$\begin{aligned} \left[ \begin{matrix} {{x}_{n+1}} \\ {{y}_{n+1}} \\ \end{matrix} \right] =\left[ \begin{matrix} 1 &{} 1 \\ 1 &{} 2 \\ \end{matrix} \right] \left[ \begin{matrix} {{x}_{n}} \\ {{y}_{n}} \\ \end{matrix} \right] \bmod H \end{aligned}$$

*Step 3*: Compression measurement.

Substitute the measurement matrix $\Phi$ generated in Section "Generation of measurement matrix" and $P_{DWT}^{'}$ into Eq. (2) to obtain the measured matrix $Y$ of size $M\times N$.

*Step 4*: Normalization.

Using the maximum value $Y_{max}$ and minimum values $Y_{min}$ of the pixels in the measurement result matrix, map the pixel values to the [0, 255] interval to obtain an intermediate image $C_{1}$ in digital image format.

12 $$\begin{aligned} {{C}_{1}}=round\left( 255\times \frac{Y-{{Y}_{\min }}}{{{Y}_{\max }}-{{Y}_{\min }}}\right) \end{aligned}$$

*Step 5*: Odd-even interleaved diffusion.

1. Construct two matrices containing only the odd pixel point locations and even pixel point locations in image $C_{1}$, respectively^47^.
  13 $$\begin{aligned} {\left\{ \begin{array}{ll} {{Q}_{1}}(a)={{C}_{1}}(2a-1) \\ {{Q}_{2}}(a)={{C}_{1}}(2a) \\ \end{array}\right. } \end{aligned}$$
  where $$a\in \{1,2,3,...,M\times W/2\}$$.
2. Diffusion matrix $C_{21}$ calculated from Eqs. (14) and (15).
  14 $$\begin{aligned}{} & {} {{C}_{21}}(1)={{Q}_{1}}(1)\oplus {{C}_{21}}(M\times W/2)\oplus {{R}_{1}}(1) \end{aligned}$$
  15 $$\begin{aligned}{} & {} {\left\{ \begin{array}{ll} {{C}_{21}}(a)={{Q}_{1}}(a)\oplus {{C}_{21}}(a-1)\oplus {{R}_{1}}(a),\textit{ a is an odd number} \\ {{C}_{21}}(a)={{Q}_{2}}(a)\oplus {{C}_{21}}(a-1)\oplus {{R}_{1}}(a),\textit{ a is an even number} \\ \end{array}\right. } \end{aligned}$$
3. Diffusion matrix $C_{22}$ calculated from Eqs. (16) and (17).
  16 $$\begin{aligned}{} & {} {{C}_{22}}(1)={{Q}_{2}}(1)\oplus {{C}_{22}}(M\times W/2)\oplus {{R}_{2}}(1) \end{aligned}$$
  17 $$\begin{aligned}{} & {} {\left\{ \begin{array}{ll} {{C}_{22}}(b)={{Q}_{2}}(b)\oplus {{C}_{22}}(b-1)\oplus {{R}_{2}}(b),\textit{ b is an odd number} \\ {{C}_{22}}(b)={{Q}_{1}}(b)\oplus {{C}_{22}}(b-1)\oplus {{R}_{2}}(b),\textit{ b is an even number} \\ \end{array}\right. } \end{aligned}$$
  where $$b\in \{1,2,3,...,M\times W/2\}$$.

*Step 6*: Bit-level row and column permutation.

Generate the corresponding sort indexes $index_{3}$ and $index_{4}$ according to the chaotic sequences $R_{3}$ and $R_{4}$. Convert each pixel point in the diffused image $C_{2}$ to an 8-bit binary number, newly named $C_{2\_BIT}$ and perform the row-column permutation operation on $C_{2\_BIT}$ as follows:

18 $$\begin{aligned} {{C}_{3\_BIT}}(i,j)={{C}_{2\_BIT}}(inde{{x}_{3}}(i),inde{{x}_{4}}(j)) \end{aligned}$$

where $$i=[1,2,3,...,M\times 8]$$ and $$j=[1,2,3,...,W\times 8]$$.

Finally, the binary image $C_{3\_BIT}$ is converted to decimal format to obtain the final ciphertext image $C$.

### Decryption step

Image decryption is the inverse process of encryption. Taking the cipher image $C$ as input, the decryption process is briefly described as follows:

*Step 1*: Decryption of row and column permutation.

The same method to get the index sequence $index_{3}$ and $index_{4}$, then convert the cipher image $C$ to binary format $C_{3\_BIT}$. The decryption process of row-column permutation is as follows:

19 $$\begin{aligned} {{C}_{2\_BIT}}(inde{{x}_{3}}(i),inde{{x}_{4}}(j))={{C}_{3\_BIT}}(i,j) \end{aligned}$$

*Step 2*: Decryption of odd-even interleaved diffusion.

Similar to the encryption process, sequences $R_{1}$ and $R_{2}$ are obtained using Eq. (7). After converting the inverse permutated image $C_{2\_BIT}$ to decimal format image $C_{2}$, decryption is performed through odd-even interleaved diffusion using the following formula:

20 $$\begin{aligned}{} & {} {\left\{ \begin{array}{ll} {{Q}_{1}}(a)={{C}_{21}}(a)\oplus {{C}_{21}}(a-1)\oplus {{R}_{1}}(a),\textit{ a is an odd number} \\ {{Q}_{2}}(a)={{C}_{21}}(a)\oplus {{C}_{21}}(a-1)\oplus {{R}_{1}}(a),\textit{ a is an even number} \\ \end{array}\right. } \end{aligned}$$

21 $$\begin{aligned}{} & {} {\left\{ \begin{array}{ll} {{Q}_{2}}(b)={{C}_{22}}(b)\oplus {{C}_{22}}(b-1)\oplus {{R}_{2}}(b),\textit{ b is an odd number} \\ {{Q}_{1}}(b)={{C}_{22}}(b)\oplus {{C}_{22}}(b-1)\oplus {{R}_{2}}(b),\textit{ b is an even number} \\ \end{array}\right. } \end{aligned}$$

22 $$\begin{aligned}{} & {} {\left\{ \begin{array}{ll} {{Q}_{1}}(1)={{C}_{21}}(1)\oplus {{Q}_{1}}(M\times H/2)\oplus {{R}_{1}}(1) \\ {{Q}_{2}}(1)={{C}_{22}}(1)\oplus {{Q}_{2}}(M\times H/2)\oplus {{R}_{2}}(1) \\ \end{array}\right. } \end{aligned}$$

*Step 3*: Inverse normalization.

The inverse normalization operation is performed on the matrix $C_{2}$ to obtain the matrix $C_{1}$. The formula for this process is as follows:

23 $$\begin{aligned} {{C}_{1}}=\frac{{{C}_{2}}\times ({{Y}_{\max }}-{{Y}_{\min }})}{255}+{{Y}_{\min }} \end{aligned}$$

*Step 4*: OMP reconstruction.

Using the measurement matrix generated in Section "Generation of measurement matrix", we can reconstruct the sparsely scrambled image ${{P}_{DWT}}$ from the matrix $C_{1}$ using the OMP algorithm. This process can be represented as follows and the pseudo-code is given in algorithm 2:

24 $$\begin{aligned} P_{DWT}=omp(C_1,\Phi ,H) \end{aligned}$$

**Algorithm 2:**
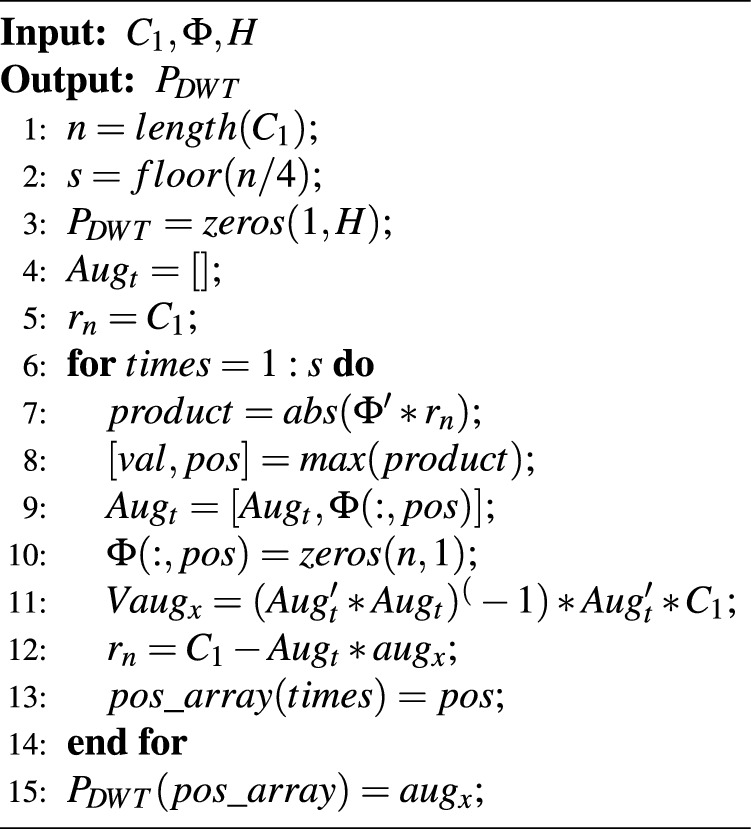
OMP reconstruction.

*Step 5*: Decryption of Arnold scrambling and inverse DWT.

Based on the definition of Arnold scrambling in Eq. (5), the reverse operation process can be deduced, allowing the recovery of the sparsely scrambled image ${{P}_{DWT}}$. Finally, applying the inverse Discrete Wavelet Transform operation to the sparse image results in the reconstruction of the image $P$ of size $H\times W$.

25 $$\begin{aligned} P=iDWT({{P}_{DWT}}) \end{aligned}$$

## Simulation results and performance analysis

### Experimental environment

We employed an 11th Gen Intel(R) Core(TM) i5-11400H CPU in a mainframe PC with MATLAB R2022a experimental software loaded as the experimental platform. A selection of experimental images was made from the USC-SIPI repository^48^.

### Statistical analytics

#### Histogram analysis

**Figure 6:**
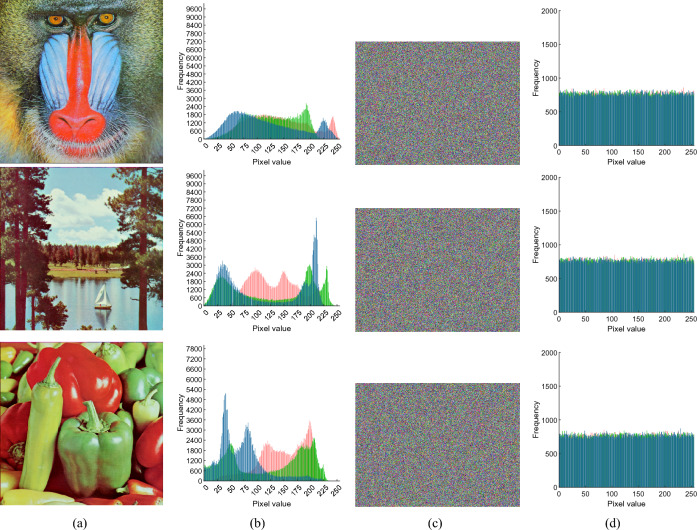
Images before and after encryption: (**a**) Original image; (**b**) Histogram of original image; (**c**) Encrypted image at CR=0.75; (**d**) Histogram of encrypted image.

**Figure 7:**
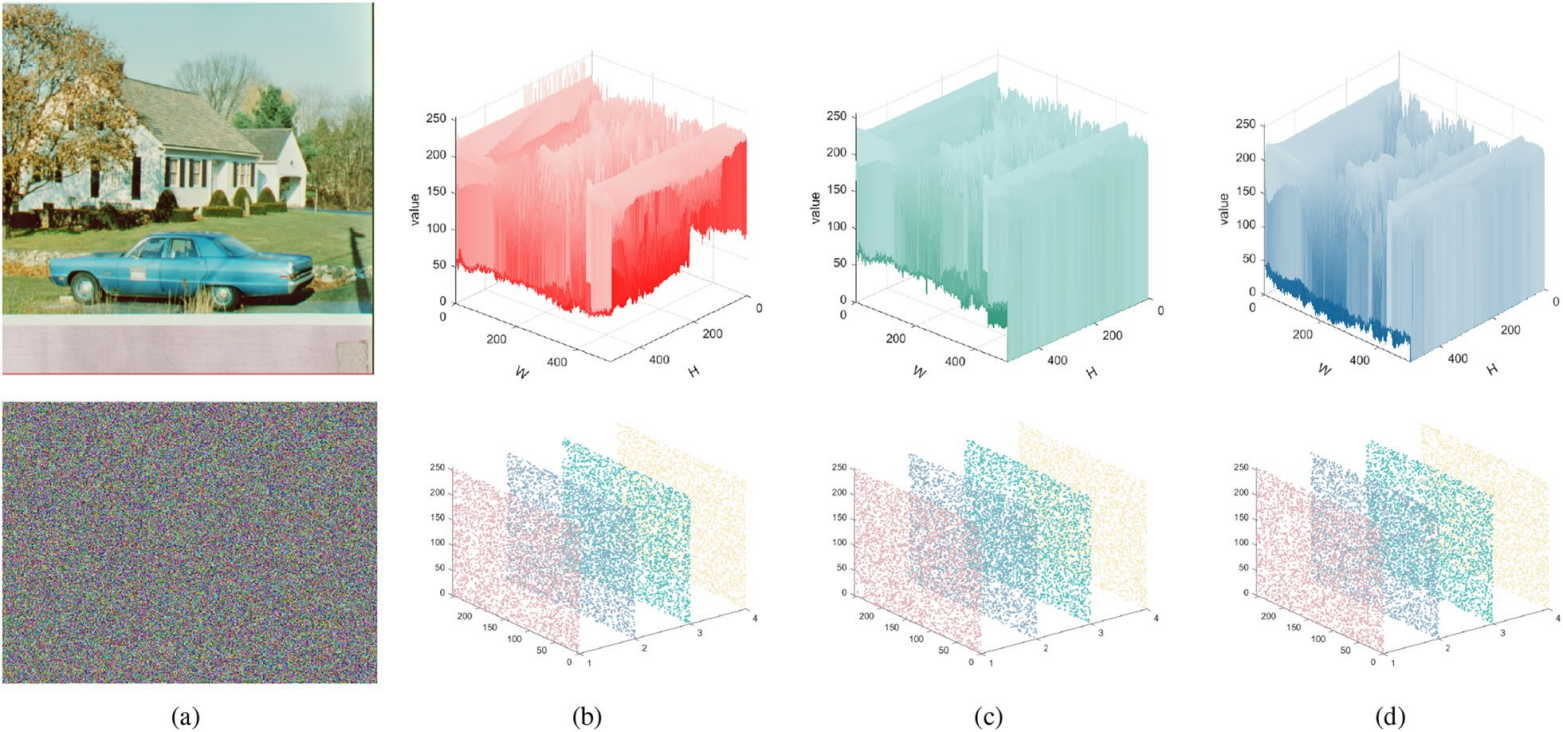
3D visualization of the encrypted image simulation shown: (**a**) plaintext image and ciphertext image; (**b**) red channel; (**c**) green channel; (**d**) blue channel.

The histogram can visualize the distribution of all pixels in the image, and for the given plain image Fig. 6a and the corresponding cipher image Fig. 6c, their 2D histograms are shown in Fig. 6b and d, respectively. Additionally, the 3D histogram corresponding to Fig. 7a is given in Fig. 7b–d, which show that the plaintext image presents a certain statistical regularity, whereas the statistical properties of the encrypted histogram of the image present a noise-like distribution, which well hides the grey-value information of the image, and thus improves the ability of resisting the attack of statistical analysis.

#### Adjacent pixel correlation analysis

**Figure 8:**
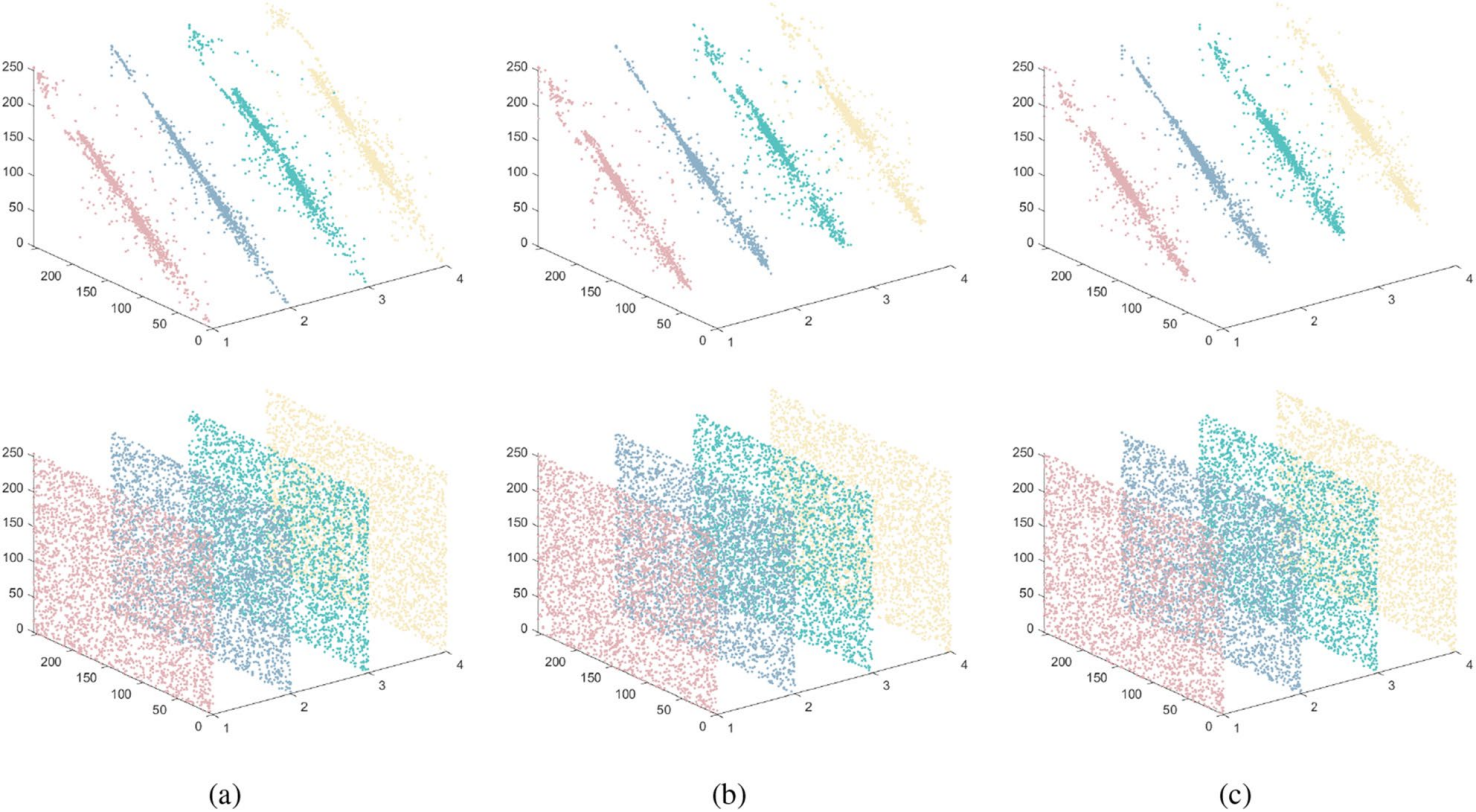
Horizontal, vertical, diagonal and antidiagonal correlation test results: (**a**) R-channel; (**b**) G-channel; (**c**) B-channel.

For plaintext picture pixels, neighboring pixel correlation is typically a notable feature, and the ciphertext through the encryption algorithm will make the adjacent pixels not associated with any pixels. This algorithm aims to generate ciphertext, in which the correlation between adjacent pixels can be ignored. The correlation coefficient calculation formula is as follows:

26 $$\begin{aligned} r_{xy}=\frac{\sum \nolimits _{i=1}^{M}{({{x}_{i}}-\frac{1}{M}\sum \nolimits _{j=1}^{M}{{{x}_{j}}})({{y}_{i}}-\frac{1}{M}\sum \nolimits _{j=1}^{M}{{{y}_{j}}})}}{\sqrt{\sum \nolimits _{i=1}^{M}{{{({{x}_{i}}-\frac{1}{M}\sum \nolimits _{j=1}^{M}{{{x}_{j}}})}^{2}}}}\sqrt{\sum \nolimits _{i=1}^{M}{{{({{y}_{i}}-\frac{1}{M}\sum \nolimits _{j=1}^{M}{{{y}_{j}}})}^{2}}}}} \end{aligned}$$

where $x_{i}$ and $y_{i}$make up the first pair of adjacent pixels that are horizontal, vertical, diagonal, or anti-angle. *M* is the total number of pixels. In order to visualize the correlation between adjacent pixels in plaintext and ciphertext, we calculate and compare the difference between the two, as shown in Fig. 8. It appears that the plaintext pixels have a significant degree of association, but the ciphertext pixels show almost no correlation at all. This finding demonstrates how robust the method is against statistical attacks.

#### Differential statistical analysis

The difference between the two images can be quantified using two criteria: The Unified Average Changed Intensity (UACI) and the Number of Pixels Change Rate (NPCR) are the measures of interest. In differential attacks, attackers often make slight changes to the plaintext image, using a specific algorithm to encrypt it before and after the adjustments, aiming to reveal their relationship. The explanation of UACI and NPCR is as follows:

27 $$\begin{aligned} {\left\{ \begin{array}{ll} NPCR={1\over {H\times {W}}}\times {\sum _{i=1}^H\sum _{i=1}^W D(i,j)\times 100\%}\\ UACI={1\over {H\times {W}}}{\times {\sum _{i=1}^{H}\sum _{j=1}^{W} \frac{|v_1(i,j)-v_2(i,j)|}{255}}}\times {100\%} \end{array}\right. } \end{aligned}$$

where $v_{1}$, $v_{2}$ respectively for plaintext image change a pixel before and after the ciphertext image. *D* has the following definition:

28 $$\begin{aligned} D= {\left\{ \begin{array}{ll} 0,v_1(i,j)=v_2(i,j)\\ 1,v_0(i,j)\ne {v_2(i,j)}\\ \end{array}\right. } \end{aligned}$$

Table 1 presents the NPCR and UACI values of various picture sizes that have been encrypted using the algorithm. These outcomes demonstrate the algorithm’s strong encryption capabilities.

**Table 1:** The value of NPCR and UACI.

Pictures	Description	Size	Type	NPCR($\%$)		UACI($\%$)	
				Ours	LICM-IEA^49^	Ours	LICM-IEA^49^
5.1.09	Moon surface	256	Gray	99.6412	99.6126	33.4232	33.4029
5.1.10	Aerial	256	Gray	99.5988	99.6184	33.4122	33.3627
4.1.01	Female	256	Color	99.5941	99.6027	33.5322	33.4625
4.1.02	Couple	256	Color	99.6123	99.6238	33.3684	33.4221
7.1.01	Truck	512	Gray	99.5689	99.5991	33.4012	33.3977
7.1.02	Airplane	512	Gray	99.6211	99.5899	33.4972	33.3386
2.1.09	San Diego(Point Loma)	512	Color	99.6321	99.6381	33.3979	33.4123
2.1.10	San Diego(Shelter Island)	512	Color	99.6411	99.6128	33.4265	33.4235
7.2.01	Airplane(U-2)	1024	Gray	99.6040	99.6352	33.4188	33.4266
2.1.10	San Francisco(Bay Bridge)	1024	Color	99.6132	99.5901	33.3979	33.4115

#### Entropy of information

In order to assess the distribution of gray values in images and measure the degree of unpredictability in picture data, information entropy is essential. This is its definition:

29 $$\begin{aligned} H(x)=-\sum _{i=1}^{L}P(x_i)log_2{P(x_i)} \end{aligned}$$

where *x* denotes the pixel value and $p(n_{x})$ denotes the probability of the symbol. Taking the pixel value of 8 bits as an example, the theoretical value is 8. It can be seen from Table 2 that the experimental results are very close to 8, indicating that the algorithm has good information entropy characteristics.

**Table 2:** Plaintext and ciphertext information entropy of different images.

Pictures	Description	Size	Type	Plain image	Cipher image	LICM-IEA^49^
4.1.03	Female(from Bell Labs)	256	Color	5.9709	7.9986	7.9998
4.1.05	House	256	Color	7.0686	7.9988	7.9986
4.2.05	Airplane(F-16)	512	Color	6.6639	7.9997	7.9985
4.2.06	Sailboat on lake	512	Color	7.7622	7.9997	7.9981
2.2.07	Oakland	1024	Color	6.4492	7.9999	7.9989
2.2.12	San Francisco and Oakland	1024	Color	6.9619	7.9999	7.9992

### Recovery image quality analysis

The mean square error (MSE) of the encrypted image’s plaintext and ciphertext, as well as its db expression (PSNR) in signal processing, are used in this experiment. The expression is as follows:

30 $$\begin{aligned} P S N R=10 \times \log _{10}\left( \frac{Q^2}{(1 / H W) \sum _{i=1}^H \sum _{j=1}^W[X(i, j)-Y(i, j)]^2}\right) \end{aligned}$$

where *Q* represents the pixel level of the image.

For digital images, the PSNR value higher than 40 dB indicates that the image quality is good. Table 3 displays the testing results and Table 4 demonstrates the comparison results of the proposed algorithm with other advanced algorithms, both of which prove the high recovery quality of the algorithm.

**Table 3:** Image recovery quality analysis of different images under CR=0.5.

Pictures	Description	Size	Type	PSNR(dB)
5.1.09	Moon surface	256	Gray	39.4465
5.1.11	Airplane	256	Gray	42.9522
4.1.03	Female(from Bell Labs)	256	Color	44.0599
4.1.05	House	256	Color	42.4047
4.2.05	Aerial	512	Gray	38.0820
4.2.05	Airplane(F-16)	512	Color	41.4172
4.2.06	Sailboat on lake	512	Color	41.4166
2.2.07	Oakland	1024	Color	40.3476
2.2.12	San Francisco and Oakland	1024	Color	39.3316

**Table 4:** Comparison results on PSNR between the recovered image and plain image.

Algorithms	Compression ratio		
	0.25	0.5	0.75
Ours	32.7133	39.5172	42.3821
^50^	–	23.3608	34.7149
^51^	32.3566	36.1892	37.4529
^52^	30.1022	36.8294	41.2933

### Impact of different key parameters on recovery quality

In the image encryption algorithm based on compressive sensing, there are several key parameters that affect its reconstruction quality, such as compression rate, reconstruction algorithm, sparse representation method, etc. In this section we aim to show the impact of different reconstruction algorithms and three commonly used sparse representation methods on the recovery quality. The test image is a picture of jelly beans taken at USC. of size $256\times 256$. The specific two sections are as follows.

#### Impact of reconstruction algorithms on recovery quality

**Figure 9:**
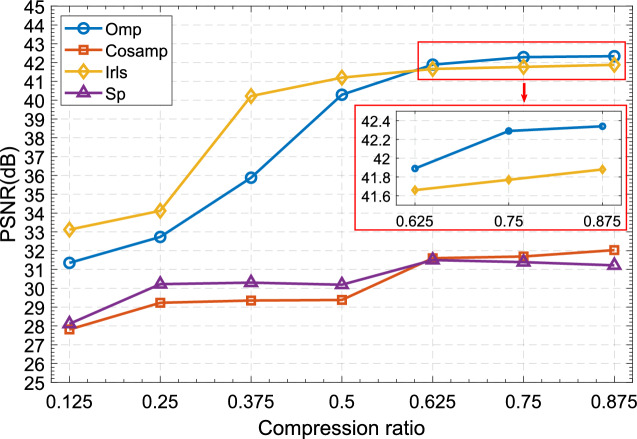
PSNR of recovered images with different reconstruction algorithms.

First, we analyze the impact of reconstruction algorithms on restored image quality under different compression rates. It can be seen from the Fig. 9 that both the OMP algorithm and the IRLS algorithm have good performance in reconstructing the measurement image. When the compression rate is only 0.125, the PSNR value of both has reached more than 30dB, and when the compression rate is 0.5 , both PSNRs reach 40dB. This shows that the size of the secret image transmitted to the receiver through the channel only needs to be half that of the ordinary algorithm, and the recovered image obtained by decryption has a very excellent recovery effect and looks the same as the original image to the naked eye.

#### Impact of different sparse representations on recovery quality

**Figure 10:**
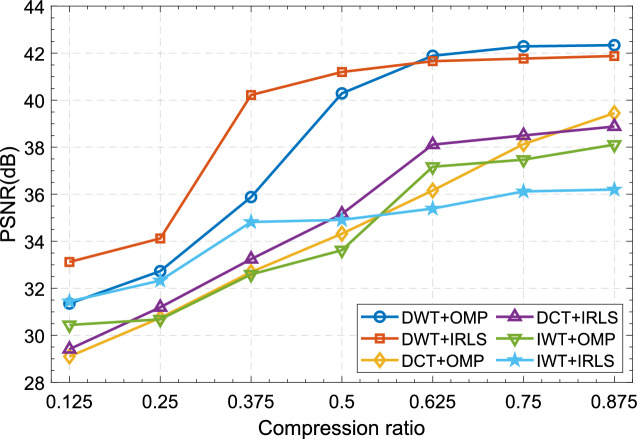
PSNR of recovered images with different sparse representations in combination with different reconstruction algorithms.

**Figure 11:**
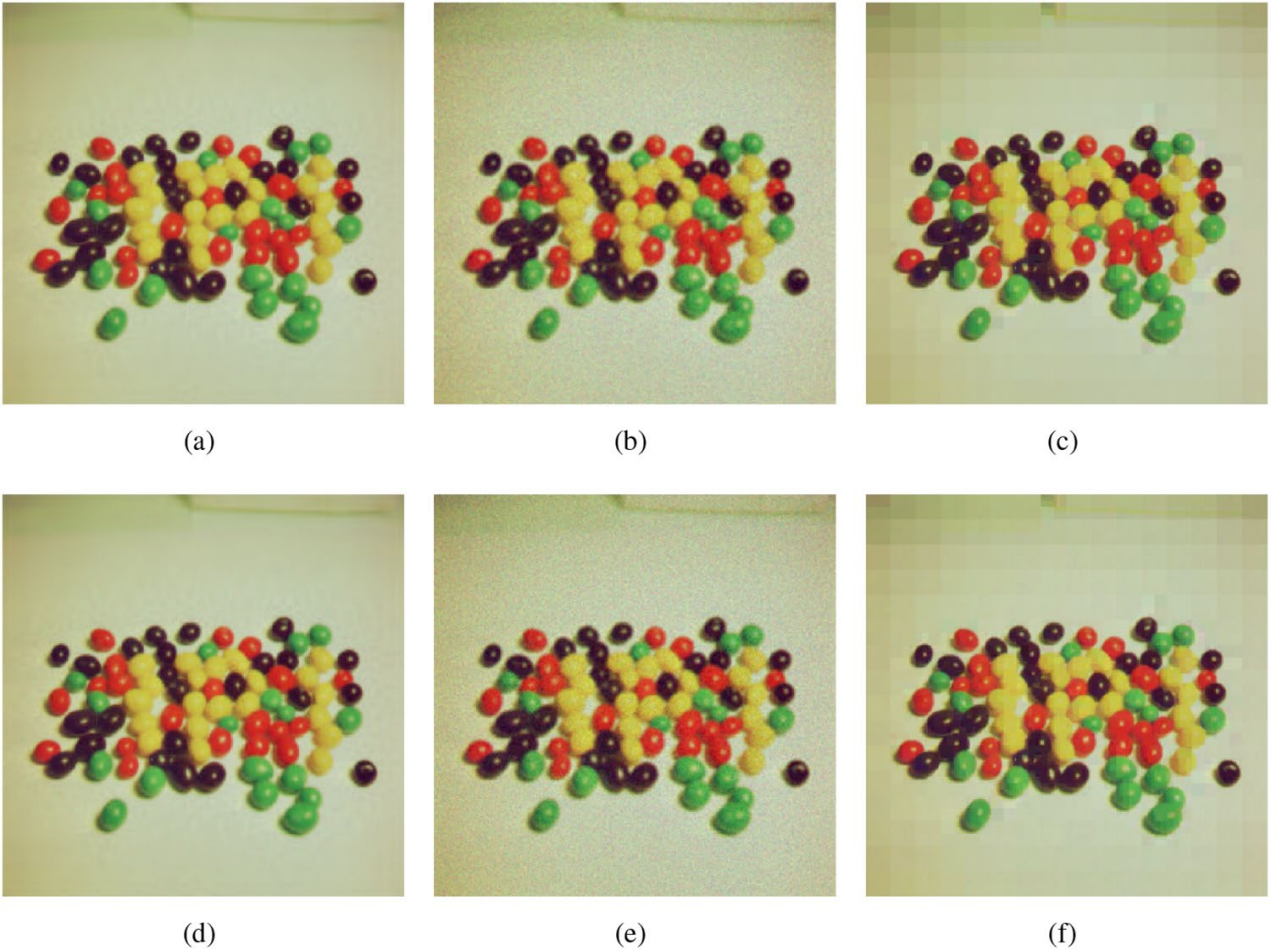
Reconstructed images under different sparse representations at CR = 0.5: (**a**) DWT+OMP; (**b**) DCT+OMP; (**c**) IWT+OMP; (**d**) DWT+IRLS; (**e**) DCT+IRLS; (f) IWT+IRLS.

The results in Section "Impact of reconstruction algorithms on recovery quality" show that the OMP and IRLS algorithms have outstanding contributions to image reconstruction, so in this section we choose these two as reconstruction algorithms to analyze three common sparse representation methods: Discrete Wavelet Transform, Integer Wavelet Transform and the effect of Discrete Cosine Transform on the quality of restored images. The test results are shown in Fig. 10. At the same time, Fig. 11 shows the restored images under the combination of different sparse representation methods and different reconstruction algorithms when the compression rate is 0.5. The experimental results show that the three sparse representation methods all have good restoration performance effects. The choice of sparse method has little impact on reconstruction, but in comparison, it is not difficult to see that Discrete Wavelet Transform has better numerical statistical results and visual performance effects. Therefore, it is reasonable to choose Discrete Wavelet Transform as the sparse representation method in this scheme. Table 5 show the recovered images of the proposed algorithm at different compression ratios.

**Table 5:**
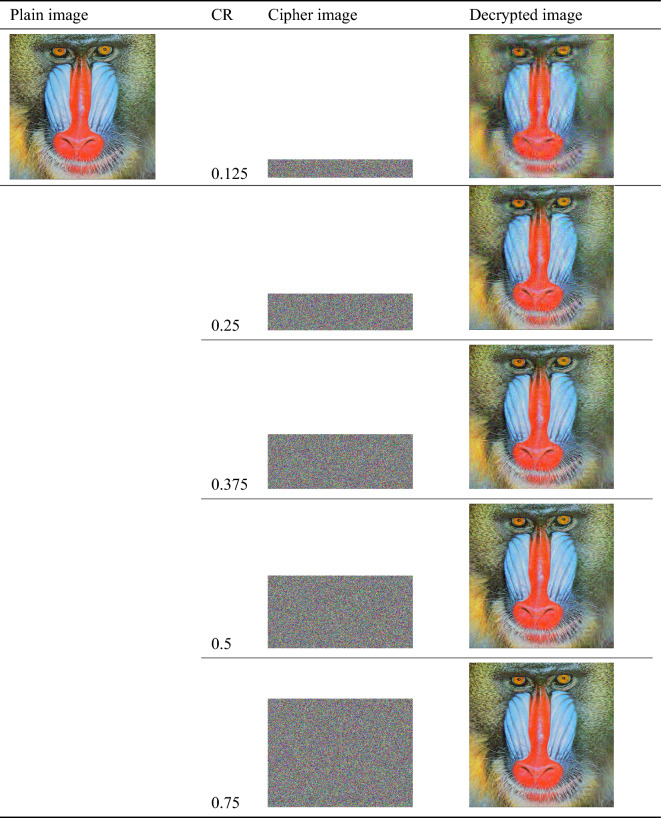
Recovery quality of proposed algorithm at different CR.

### Key space

The 2D-LSCM chaotic system used in this algorithm has a key space of $\left\{ n,m,z,SHA-256\right\}$, where the accuracy of *n*, *m*, *z* is $10^{-16}$, and $SHA-256$ is a hash of 256 bits. It can be obtained that the key space of this algorithm is about ${{10}^{3\times 16}}\times {{2}^{256}}\approx {{2}^{415}}$, and the key length reaches 415 bits. Compared with other literature, as shown in Table 6, this algorithm can resist any form of violent attack.

**Table 6:** Key space.

Papers	Key space value
Proposed method	287
^53^	154
^54^	166
^18^	224
^20^	234

#### Key sensitivity analysis

**Figure 12:**
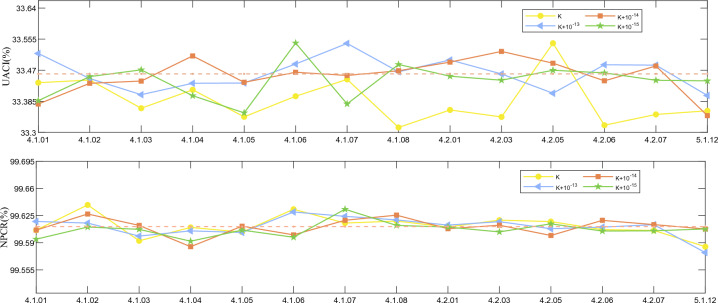
Key sensitivity test results.

Analyzing the ciphertext generated by encrypting the same picture with two slightly different keys is known as key sensitivity. This section will contrast the encryption using the proper key with the use of a slightly different key (add $\text {1}{{\text {0}}^{\text {-12}}}$, $\text {1}{{\text {0}}^{\text {-13}}}$, $\text {1}{{\text {0}}^{\text {-14}}}$ and $\text {1}{{\text {0}}^{\text {-15}}}$). The results of the analysis are shown in Fig. 12. We find that the average values of UACI and NPCR are 33.456% and 99.6105%, respectively, after adding perturbations to the key. This implies that there is a significant difference between the two cipher pictures.

#### Analysis of explicit sensitivities

**Figure 13:**
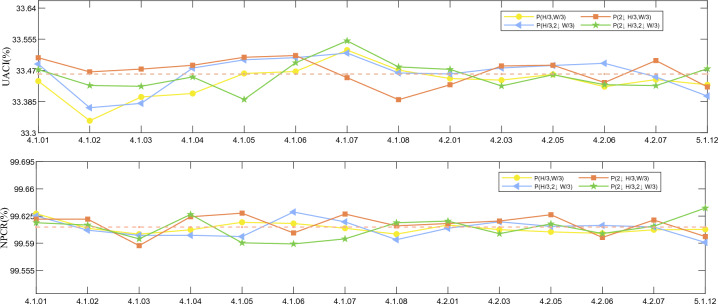
Plaintext sensitivity test results.

This section will examine how sensitive the method is to plaintext changes by setting the value of a single pixel in a common picture to 1. The pixel values at $( H / 3, W / 3 ), ( 2\times H / 3, W / 3 ), ( H / 3 , 2\times W / 3 )$ and $( 2\times H / 3, 2\times W / 3 )$ are added with 1 for comparing the size of the difference. The analysis results are presented in the accompanying Fig. 13. As can be seen from the graph, NPCR and UACI are close to the ideal values of 99.62% and 33.41%, respectively. This shows that the proposed algorithm is sufficient to resist plaintext attacks.

### Conclusion

This paper proposes a compressive sensing image encryption scheme based on optimized orthogonal measurement matrix. The algorithm employs discrete wavelet transform for a sparse representation of the image, and through Arnold scrambling, effectively reduces the correlation between adjacent pixels. After compressive sensing measurement, the odd-even interleaved diffusion and bit-level row and column permutation are performed respectively. The experimental results show that the PSNR of the restored image under the conventional compression size is above 40 dB, which indicates that this scheme has higher recovery performance than other advanced compressive sensing algorithms. Additionally, the scheme incorporates a plaintext correlation mechanism, demonstrating strong robustness against various cryptanalysis methods such as chosen plaintext attacks and differential attacks. This feature enables the scheme to effectively withstand different approaches to cryptographic analysis. A comprehensive analysis indicates that the method proposed in this paper is considered effective in enhancing the accuracy and reliability of information exchange, particularly in the context of the big data era, where it holds significant implications for image encryption.

Despite the progress made, there are still some limitations. During the experiments, we observed that for images with a compression ratio lower than 0.25, particularly those with low adjacent pixel correlation, manual adjustment of specific parameters was necessary to achieve satisfactory reconstruction results. Since parameter selection is closely tied to the image type, this process could significantly increase the cost of practical applications. In future work, we plan to conduct a thorough comparative analysis of the performance of algorithms in image reconstruction, such as coded compression and block compressive sensing. We will explore whether techniques like edge detection could be used to assess pixel correlation, automatically identify image types, and accordingly adapt the encryption parameters to ensure optimal image recovery. Moreover, the encryption framework will be further refined, enhancing both the algorithm’s security and efficiency.

## Data Availability

The datasets used and analysed during the current study available from the corresponding author on reasonable request. All data generated or analysed during this study are included in this published article.

## References

[1] Ding Y, Liu W, Wang H, Sun K (2023). A new class of discrete modular memristors and application in chaotic systems. Eur. Phys. J. Plus.

[2] Liu X, Sun K, Wang H, He S (2023). A class of novel discrete memristive chaotic map. Chaos Solitons Fractals.

[3] Gao Z (2022). Experimental demonstration of synchronous privacy enhanced chaotic temporal phase en/decryption for high speed secure optical communication. Opt. Express.

[4] Huang X, Dong Y, Ye G, Shi Y (2022). Meaningful image encryption algorithm based on compressive sensing and integer wavelet transform. Front. Comput. Sci..

[5] Yuan X, Cai Z (2022). Ichv: A new compression approach for industrial images. IEEE Trans. Ind. Inform..

[6] Erkan U, Toktas A, Lai Q (2023). 2d hyperchaotic system based on Schaffer function for image encryption. Expert Syst. Appl..

[7] Zou C, Wang X, Zhou C, Xu S, Huang C (2022). A novel image encryption algorithm based on DNA strand exchange and diffusion. Appl. Math. Comput..

[8] Feng W, Qin Z, Zhang J, Ahmad M (2021). Cryptanalysis and improvement of the image encryption scheme based on Feistel network and dynamic DNA encoding. IEEE Access.

[9] Lu D, Li M, Liao Y, Tao G, Cai H (2023). Verifiable privacy-preserving queries on multi-source dynamic DNA datasets. IEEE Trans. Cloud Comput..

[10] Teng L, Wang X, Yang F, Xian Y (2021). Color image encryption based on cross 2d hyperchaotic map using combined cycle shift scrambling and selecting diffusion. Nonlinear Dyn..

[11] Lai Q, Hu G, Erkan U, Toktas A (2023). A novel pixel-split image encryption scheme based on 2d Salomon map. Expert Syst. Appl..

[12] Wu T (2023). Secure turbulence-resistant coherent free-space optical communications via chaotic region-optimized probabilistic constellation shaping. Opt. Lett..

[13] Ye X, Zhang Y, Xiao X, Yi S, Lan R (2023). Usability enhanced thumbnail-preserving encryption based on data hiding for jpeg images. IEEE Signal Process. Lett..

[14] Zhou W, Zhang Y, Zhao R, Yi S, Lan R (2023). Adversarial thumbnail-preserving transformation for facial images based on GAN. IEEE Signal Process. Lett..

[15] Ma Y, Chai X, Gan Z, Zhang Y (2023). Privacy-preserving TPE-based jpeg image retrieval in cloud-assisted internet of things. IEEE Internet Things J..

[16] Wen H (2022). Secure DNA-coding image optical communication using non-degenerate hyperchaos and dynamic secret-key. Mathematics.

[17] Chen X, Mou J, Cao Y, Yan H, Jahanshahi H (2023). A chaotic color image encryption scheme based on improved Arnold scrambling and dynamic DNA encoding. Multimed. Tools Appl..

[18] Wen H, Kang S, Wu Z, Lin Y, Huang Y (2023). Dynamic RNA coding color image cipher based on chain feedback structure. Mathematics.

[19] Luo Y, Liang Y, Zhang S, Liu J, Wang F (2022). An image encryption scheme based on block compressed sensing and Chen’s system. Nonlinear Dyn..

[20] Wen H, Huang Y, Lin Y (2023). High-quality color image compression-encryption using chaos and block permutation. J. King Saud Univ. Comput. Inf. Sci..

[21] Huang H, Cai Z (2023). Duple color image encryption system based on 3-d nonequilateral Arnold transform for IIot. IEEE Trans. Ind. Inform..

[22] Lu Q, Liao X, Xiang T, Li H, Huang T (2021). Privacy masking stochastic subgradient-push algorithm for distributed online optimization. IEEE Trans. Cybern..

[23] Erkan U, Toktas A, Memiş S, Lai Q, Hu G (2023). An image encryption method based on multi-space confusion using hyperchaotic 2d Vincent map derived from optimization benchmark function. Nonlinear Dyn..

[24] Feng W (2022). Image encryption algorithm based on plane-level image filtering and discrete logarithmic transform. Mathematics.

[25] Feng W, Zhang J, Qin Z (2021). A secure and efficient image transmission scheme based on two chaotic maps. Complexity.

[26] Wen H (2023). Secure optical image communication using double random transformation and memristive chaos. IEEE Photonics J..

[27] Lai Q, Yang L, Liu Y (2022). Design and realization of discrete memristive hyperchaotic map with application in image encryption. Chaos, Solitons Fractals.

[28] Zhou S, Qiu Y, Qi G, Zhang Y (2023). A new conservative chaotic system and its application in image encryption. Chaos, Solitons Fractals.

[29] Zhou S, Wang X, Zhang Y (2023). Novel image encryption scheme based on chaotic signals with finite-precision error. Inf. Sci..

[30] Liu, W., Sun, K., He, S. & Wang, H. The parallel chaotification map and its application. *IEEE Trans. Circuits Syst. I Regular Pap.* 1–10 (2023).

[31] Kocak O, Erkan U, Toktas A, Gao S (2024). Pso-based image encryption scheme using modular integrated logistic exponential map. Expert Syst. AppL..

[32] Wen H, Lin Y, Kang S, Zhang X, Zou K (2023). Secure image encryption algorithm using chaos-based block permutation and weighted bit planes chain-diffusion. iScience.

[33] Toktas A, Erkan U, Gao S, Pak C (2024). A robust bit-level image encryption based on Bessel map. Appl. Math. Comput..

[34] Hua Z, Liu X, Zheng Y, Yi S, Zhang Y (2023). Reversible data hiding over encrypted images via preprocessing-free matrix secret sharing. IEEE Trans. Circuits Syst. Video Technol..

[35] Wen H, Lin Y (2023). Cryptanalyzing an image cipher using multiple chaos and DNA operations. J. King Saud Univ. Comput. Inf. Sci..

[36] Luo Y, Zhang C, Wang X, Liang X, Qiu K (2023). Robust key update with controllable accuracy using support vector machine for secure Ofdma-Pon. J. Lightwave Technol..

[37] Liang X, Zhang C, Luo Y, Wang X, Qiu K (2023). Secure encryption and key management for Ofdm-Pon based on chaotic Hilbert motion. J. Lightwave Technol..

[38] Wen H, Lin Y (2024). Cryptanalysis of an image encryption algorithm using quantum chaotic map and DNA coding. Expert Syst. Appl..

[39] Wen H, Lin Y, Yang L, Chen R (2024). Cryptanalysis of an image encryption scheme using variant Hill cipher and chaos. Expert Syst. Appl..

[40] Chai X (2023). Exploiting semi-tensor product compressed sensing and hybrid cloud for secure medical image transmission. IEEE Internet Things J..

[41] Ye G, Liu M, Yap W-S, Goi B-M (2023). Reversible image hiding algorithm based on compressive sensing and deep learning. Nonlinear Dyn..

[42] Wang X, Liu C, Jiang D (2022). A novel visually meaningful image encryption algorithm based on parallel compressive sensing and adaptive embedding. Expert Syst. Appl..

[43] Wang X, Su Y (2021). Image encryption based on compressed sensing and DNA encoding. Signal Process. Image Commun..

[44] Chen Z, Ye G (2022). An asymmetric image encryption scheme based on hash sha-3, rsa and compressive sensing. Optik.

[45] Liang J (2023). A secure and effective image encryption scheme by combining parallel compressed sensing with secret sharing scheme. J. Inf. Secur. Appl..

[46] Hua Z, Jin F, Xu B, Huang H (2018). 2d logistic-sine-coupling map for image encryption. Signal Process..

[47] Wang X, Wang Y (2023). Multiple medical image encryption algorithm based on scrambling of region of interest and diffusion of odd-even interleaved points. Expert Syst. Appl..

[48] The USC-SIPI image database. https://sipi.usc.edu/database/.

[49] Cao C, Sun K, Liu W (2018). A novel bit-level image encryption algorithm based on 2d-LICM hyperchaotic map. Signal Process..

[50] Wang X (2021). Image encryption based on compressed sensing and DNA encoding. Signal Process. Image Commun..

[51] Chai X (2020). An efficient visually meaningful image compression and encryption scheme based on compressive sensing and dynamic lsb embedding. Optics Lasers Eng..

[52] Zhang C (2023). Plaintext-related image encryption scheme without additional plaintext based on 2dcs. Optik.

[53] Mansouri A, Wang X (2021). A novel block-based image encryption scheme using a new sine powered chaotic map generator. Multimed. Tools Appl..

[54] Su Y, Wang X, Xu M, Zou C, Liu H (2023). A three-dimensional (3d) space permutation and diffusion technique for chaotic image encryption using Merkel tree and dna code. Sens. Imaging.

