# Author Correction: Exploiting high‑quality reconstruction image encryption strategy by optimized orthogonal compressive sensing

**DOI:** 10.1038/s41598-024-61602-5

**Published:** 2024-05-23

**Authors:** Heping Wen, Lincheng Yang, Chixin Bai, Yiting Lin, Tengyu Liu, Lei Chen, Yingchun Hu, Daojing He

**Affiliations:** 1 Zhongshan Institute, University of Electronic Science and Technology of China, Zhongshan, 528402 China; 2 School of Information and Communication Engineering, University of Electronic Science and Technology of China, Chengdu, 611731 China; 3 School of Computer Science and Technology, Harbin Institute of Technology (Shenzhen), Shenzhen, 518118 China; 4 GuangDong Engineering Technology Research Center of Cryptographic Product and System Evaluation, Shenzhen, 518118 China

Correction to: *Scientific Reports* 10.1038/s41598-024-59277-z, published online 16 April 2024

The Acknowledgements section in the original version of this Article was incomplete.

“This work was supported in part by Guangdong Basic and Applied Basic Research Foundation under Grant 2023A1515011717, and in part by Project for Zhongshan Science and Technology under Grant 2021B2062, and in part by Special Projects for Key Fields of the Education Department of Guangdong Province under Grant 2023ZDZX1041.”

now reads:

“This work was supported in part by Guangdong Basic and Applied Basic Research Foundation under Grant 2023A1515011717, and in part by Project for Zhongshan Science and Technology under Grant 2021B2062, and in part by Special Projects for Key Fields of the Education Department of Guangdong Province under Grant 2023ZDZX1041, and in part by the National Natural Science Foundation of China under Grant 62376074, and in part by the Shenzhen Science and Technology Program under Grants SGDX20230116091244004, KCXST20221021111404010, JSGGKQTD20221101115655027, KJZD20230923114405011, JSGG20220831103400002, KCXFZ20230731093001002, RKX20231110090859012.”

The original Article has been corrected.

